# Extreme Anæsthesia for Obstetric Operations

**Published:** 1884-03

**Authors:** J. MacF. Gaston

**Affiliations:** Atlanta, Ga.


THE ATLANTA

MEDICAL AND SURGICAL JOURNAL.

VOL. I. MARCH, 1884. No. 1

### Original Communications.

### EXTREME ANÆSTHESIA FOR OBSTETRIC OPERATIONS

BY J. MACF. GASTON, M.D., ATLANTA, GA.

The inhalation of chloroform in labor is ordinarily attended with insensibility to pain, without arresting the uterine contractions, and is found advantageous in relaxing the external soft parts, so as to facilitate the delivery of the child. But in the management of some complicated cases of labor, the overpowering effect of the continued use of inhalations of chloroform has reached the point of interrupting completely the contractions of the womb, and left its walls in such a state of relaxation as to be attended with a flaccidity in grasping the outline of the womb through the abdominal walls. Such a result was observed for the first time a few years ago, and the general condition of the patient did not present any features worthy of special note apart from the profound narcosis. It was a first labor, with an impracticable face presentation, in which she had been kept under the profound influence of chloroform for more than an hour, when, in the course of the efforts with the hand and the lever to correct the position, it was found that the entire head receded from the vulva. A colleague who was in attendance with me then verified that all uterine contraction had ceased ; and upon pressing upward against the face of the child, the head was readily carried back within the womb. The hand could now be passed freely aroand the body of the child as it lay in the loose sac of the relaxed womb, and seizing the feet, there was no difficulty whatever in effecting podalic version and delivering the dead child. The chloroform had been suspended immediately upon observing the complete arrest of the contractility of the fibres of the muscular structure of the uterine walls, and before the extraction was effected a return of energy was noted that coöperated in expelling the head. It was evident that the influence of the anæsthetic had extended beyond the control of the animal or voluntary system of nerves, and had reached in its effect the functions of the organic nervous distribution to the womb. The automatic contraction of the womb, dependent upon the ganglionic supply of nerves, which continues under the moderated impression of chloroform, ceased entirely under a more profound influence of this anæsthetic. This result being accompanied with no serious shock to the recuperative powers of the patient, I was led to reflect that extreme anæsthesia might be available for the relaxation of the womb, not alone in impracticable face presentations, but in all mal-positions of the child, requiring interference on the part of the obstetrician. It is well known to all, who have been called upon to correct shoulder presentations under clonic contractions of the womb, how little benefit is derived from the ordinary mode of administering inhalations of chloroform or ether; and this case goes to show that a more complete narcosis will insure entire relaxation.

A favorable opportunity was offered some months ago for testing this practice in another case of face presentation under the care of two colleagues, who sought my assistance, after exhausting their resources without effect. I explained to them what had resulted in the case referred to above, and suggested that it might avail in the emergency, in which they acquiesced, and coöperated by administering the chloroform very freely, so that the woman was speedily brought under its profound anæsthetic influence, indicated by stertorious breathing. At this stage of its effect, and not previously, it was perceived that the rigid contraction of the uterine walls relaxed, and upon making considerable pressure upon the face of the child that was presenting at the outlet of the inferior strait, the head was dislodged from its impacted position, and carried without further difficulty up through the superior strait into the womb. At this moment there was a gush of blood from the vulva, such as I had never witnessed in a considerable experience, with hemorrhages from the womb; and realizing that there was no time to be lost, the hand was hooked over the occiput so as to bring the chin round against the breast, and by traction the vertex presentation was restored, so as to bring the head at once into the superior strait, thus serving as a tampon. The chloroform was stopped, and cold water dashed over the abdomen to excite the contractility to the uterine fibres, and forceps being applied, the child was, with strong traction by myself, alternated by one of my colleagues, eventually delivered dead. There was no further hemorrhage after the delivery, and the womb contracted regularly.

The hemorrhage encountered was doubtless from the partial detachment of the placenta, leaving the patulous openings on the inner surface of the womb free, when the entire relaxation of the organ occurred.

I have purposely refrained from giving any of the minute details of these unfavorable presentations of the face, which are among the most difficult to manage, upon the principle heretofore adopted, so as to draw the special attention of obstetricians to the interruption of the uterine contraction by the extreme influence of the anæsthetic. A case of face presentation that cannot by any means at our command be brought into a position for delivery may readily be forced back into the cavity of the womb, when it becomes relaxed and changed to a vertex presentation, or relieved of all embarrassment by turning, and thus in other cases of dystocia.

It is not claimed that a rule can be established upon the limited observation of the result in two cases, but such an effect has not within my range of reading been noted previously, and a knowledge of this fact may open the way to important steps in correcting all mal-positions of the child accompanied by clonic uterine contractions.

Upon further experience it may also be discovered that extreme anæsthesia will relax the hour-glass and other irregular contractions of the uterine walls, which the ordinary full inhalations do not reach, as I have verified in manual dilatations of these spasms under the use of chloroform in numerous instances.

Recent reports of the use of nitrite of amyl and bromide of ethyl encourage a trial of them in this class of cases. Fancourt Barnes, of London, has resorted to the nitrite of amyl in spasms of the womb with a satisfactory result, and others in this country have confirmed his observation of its good effect in hour-glass contraction. It is therefore to be inferred that it may relax the uterine contractions in labor.

The bromide of ethyl has also a claim to consideration as an anæsthetic in labor; but while Heckermann, of Berlin, reports favorably upon its general effects in fifty cases, Muller, with an experience of twenty cases, takes serious exceptions to the uniformity of its influence.

Among the cases submitted to the bromide of ethyl, it is stated that in five during the second stage, the labor pains ceased altogether, and in three they ceased in part, thus favoring the recourse to this agent as a means of relaxation of the uterine contractions. It may be inferred that with the cessation of pain, the uterine contractions are suspended, and if the medicine is increased, such a relaxation may be obtained as to facilitate the correction of mal-positions. "On the average fifty to sixty grains of the drug were employed. During the narcosis the pupils dilated, and the face became congested. Little effect was produced on the pulse and respiration."

The indications for such an impression upon the contractility of the uterine fibres as to stay their action for a time, which may admit of version in impracticable presentations of all varieties, are most urgent, as the obstetrician encounters the greatest difficulty frequently in his manipulations, owing to the resistance of the walls of the womb. Should a temporary relaxation of the contractions be accomplished uniformly by the extreme anæsthesia of chloroform, or by the influence of the nitrite of amyl, or bromide of ethyl, an important adjuvant will be gained in the correction of mal-positions by the obstetrician.

